# Visible-Light-Enhanced Antibacterial Activity of Silver and Copper Co-Doped Titania Formed on Titanium via Chemical and Thermal Treatments

**DOI:** 10.3390/molecules28020650

**Published:** 2023-01-09

**Authors:** Kanae Suzuki, Misato Iwatsu, Takayuki Mokudai, Maiko Furuya, Kotone Yokota, Hiroyasu Kanetaka, Masaya Shimabukuro, Taishi Yokoi, Masakazu Kawashita

**Affiliations:** 1 Graduate School of Biomedical Engineering, Tohoku University, 6-6-12 Aramaki-Aoba, Aoba-ku, Sendai 980-8579, Japan; 2 Graduate School of Dentistry, Tohoku University, 4-1, Seiryo-machi, Aoba-ku, Sendai 980-8575, Japan; 3 Institute of Materials Research, Tohoku University, 2-1-1, Katahira, Aoba-ku, Sendai 980-8577, Japan; 4 Institute of Biomaterials and Bioengineering, Tokyo Medical and Dental University, 2-3-10 Kanda-surugadai, Chiyoda-ku, Tokyo 101-0062, Japan

**Keywords:** titania, silver, copper, antibacterial activity, visible-light-responsive photocatalysis

## Abstract

Dental implants made of titanium (Ti) are used in dentistry, but peri-implantitis is a serious associated problem. Antibacterial and osteoconductive Ti dental implants may decrease the risk of peri-implantitis. In this study, titania (TiO_2_) co-doped with silver (Ag) at 2.5 at.% and copper (Cu) at 4.9 at.% was formed on Ti substrates via chemical and thermal treatments. The Ag and Cu co-doped TiO_2_ formed apatite in a simulated body fluid, which suggests osteoconductivity. It also showed antibacterial activity against *Escherichia coli*, which was enhanced by visible-light irradiation. This enhancement might be caused by the synergistic effect of the release of Ag and Cu and the generation of •OH from the sample. Dental implants with such a Ag and Cu co-doped TiO_2_ formed on their surface may reduce the risk of peri-implantitis.

## 1. Introduction

Dental implants made of titanium (Ti) are widely used in dentistry, but peri-implantitis [1,2,3,4], which has a prevalence rate of about 22% [5], is a serious problem. The incidence of peri-implantitis caused by Ti dental implants can be decreased by inducing antibacterial activity via the control of surface topology [6], the incorporation of antibacterial metals [7], or a functional layer coating [8]. One strategy for preparing antibacterial Ti dental implants is the formation of a titanium oxide (TiO_2_) layer with photocatalytic antibacterial activity [9,10,11] on their surfaces. For example, Suketa et al. reported the photocatalytic antibacterial activity of TiO_2_ film formed on Ti via plasma source ion implantation [12]. It has been reported that a TiO_2_ layer formed on Ti via chemical and thermal treatments can form apatite on its surface in a simulated body fluid (SBF) [13] and bond to living bone [14]. Therefore, Ti dental implants with TiO_2_ formed on their surfaces are expected to exhibit photocatalytic antibacterial activity as well as bone-bonding ability. However, for such Ti dental implants, the photocatalytic antibacterial activity of TiO_2_ is exhibited only under exposure to short-wavelength invisible light such as ultraviolet light, which is toxic to living organisms.

When TiO_2_ is doped with elements such as nitrogen (N) [15,16] and copper (Cu) [17,18], it can show photocatalytic activity even under visible light. Several mechanisms for the visible-light-responsive photocatalytic activity of Cu-doped TiO_2_ have been proposed, depending on the chemical state of Cu [19], such as the surface plasmon resonance effect of Cu nanoparticles [20] and electron transfer from TiO_2_ to CuO [21,22] or from Cu_2_O to TiO_2_ [23,24]. It has been reported that 650 nm light from a light-emitting diode (LED) penetrates the gingiva and activates the photosensitizer within the gingival sulcus to kill bacteria that reside around the gingival sulcus [25]. Therefore, Ti dental implants with doped TiO_2_ on their surfaces can reduce the risk of peri-implantitis with periodic or on-demand irradiation of visible light at a dental clinic. We previously prepared N-doped TiO_2_ [26,27,28,29] and Cu-doped TiO_2_ [30] on Ti and investigated their surface structure, apatite formation ability in an SBF, and antibacterial activity. However, it is necessary to improve the antibacterial activity of N-doped or Cu-doped TiO_2_. One possible approach to achieve this is to increase the N or Cu content, but our method limits the amount of N or Cu that can be doped into TiO_2_ to improve photocatalytic antibacterial activity and apatite formation ability [28,31].

Therefore, in this study, we tried to co-dope silver (Ag) and Cu into TiO_2_. The excellent antibacterial properties of Ag are expected to improve the antibacterial activity of the dental implants with or without visible-light irradiation. The antibacterial activity of samples is discussed in terms of their photocatalytic activity and the release of Ag and Cu from the samples. The present findings will contribute to the development of dental implants with antibacterial activity to prevent peri-implantitis with and without visible-light irradiation.

## 2. Results and Discussion

**Figure 1:**
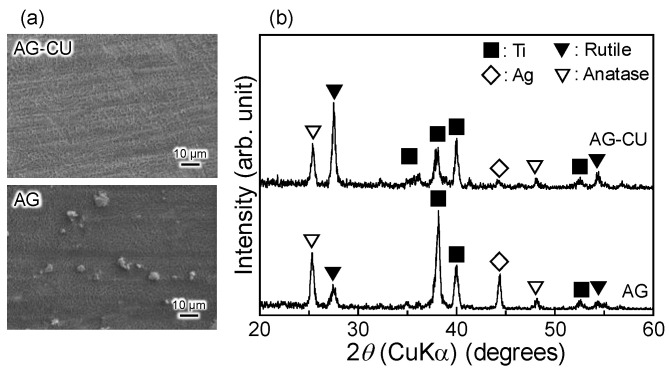
(**a**) SEM images and (**b**) TF-XRD patterns of samples.

A network-like structure formed on the surfaces of both AG-CU and AG, whereas small particles formed only on the surface of AG (Figure 1a). A similar network-like structure with small particles was previously reported [32,33,34]. The network-like structure was composed of anatase, rutile, and metallic silver (Figure 1b). The intensity of the TF-XRD peak attributed to metallic silver around the 2*θ* angle of 44° was much higher for AG than for AG-CU, which suggests that the small particles on the surface of AG were mainly composed of metallic silver. The intensity of the TF-XRD peak attributed to rutile at the 2*θ* angle of around 27° was larger than that attributed to anatase at the 2*θ* angle of around 25° for AG-CU; the opposite result was obtained for AG. This indicates that rutile and anatase preferentially formed on AG-CU and AG, respectively. The preferential formation of rutile on AG-CU was likely caused by Cu, a dopant that promotes the phase transformation of anatase to rutile [35].

AG-CU contained Ag at 2.5 at.% and Cu at 4.9 at.% on its surface, and AG contained Ag at 6.3 at.% on its surface (Table 1). AG-CU contained almost twice as much Cu as Ag. The amount of Ag in AG was higher than that in AG-CU, which can be attributed to the higher concentration of Ag in the silver nitrate (AgNO_3_) solution used for the treatment of AG (≅1 mM) compared to that (≅0.5 mM) used for the treatment of AG-CU. Although the concentrations of Ag and Cu in the AgNO_3_-Cu(NO_3_)_2_ mixed solution used for the treatment of AG-CU were the same (≅0.5 mM), AG-CU contained almost twice as much Cu as Ag on its surface. These results indicate that Ag and Cu can be co-doped into a sample by using an AgNO_3_-Cu(NO_3_)_2_ mixed solution, but the amount of Ag doped into the sample will not be simply proportional to the Ag concentration of the Ag- and Cu-containing solution used for treatment.

**Figure 2:**
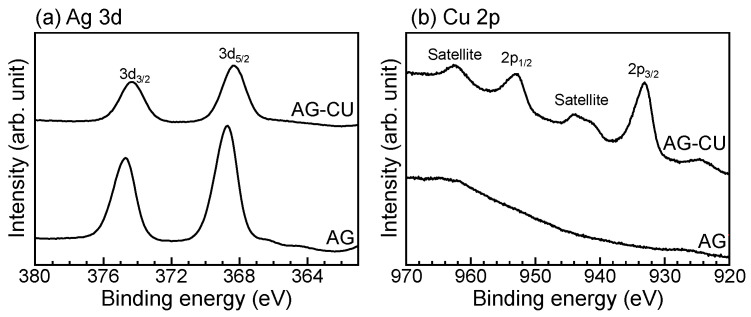
(**a**) Ag 3d and (**b**) Cu 2p electron energy region spectra of samples.

Figure 2 shows the Ag 3d and Cu 2p electron energy region spectra of the samples and Table 2 summarizes the binding energy (*E_B_*) and modified Auger parameter (α′) values of the samples. The chemical states of Ag and Cu can be determined from a comparison of *E_B_* with α′ on the Wagner plot. The Ag 3d_5/2_ peak around 368.5 eV and the α′ value of around 723.4 eV for AG-CU and AG suggest that Ag mainly existed in an oxide state on their surfaces [36]. Taking into account that the TF-XRD peaks of metallic silver were observed for AG-CU and AG (Figure 1b), we speculate that the surface of the metallic silver was oxidized in AG-CU and AG.

Cu 2p_3/2_ peaks were observed at around 933.0 eV for AG-CU, whereas no Cu 2p_3/2_ peak was observed for AG. The Cu 2p_3/2_ peak around 933.0 eV and the α’ value around 1850.4 eV suggest that Cu mainly existed as Cu_2_O on the surface of AG-CU [37]. These results indicate that copper was successfully doped into the sample surface by the present surface treatments. The lack of a TF-XRD peak corresponding to copper compounds for AG-CU (Figure 1b) and the apparent Cu 2p peak (Figure 2b and Table 1) indicate that the crystallinity of the doped copper was low for both samples. The formation of Cu_2_O with low crystallinity in AG-CU is interesting, but its mechanism is unclear. This topic is worthy of further investigation.

**Figure 3:**
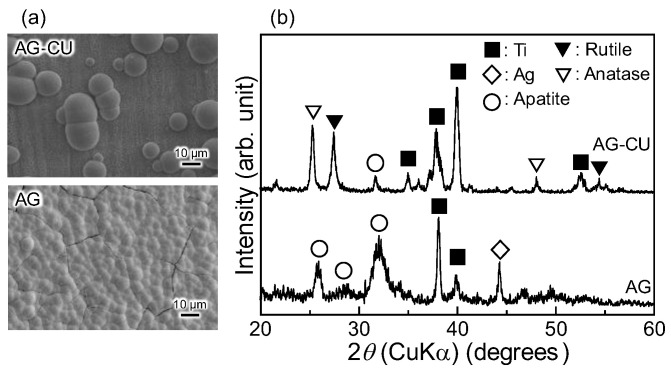
(**a**) SEM images and (**b**) TF-XRD patterns of samples after immersion in SBF for 7 days.

The SEM images of the samples after immersion in the SBF (Figure 3) indicate that apatite uniformly formed on the surface of AG, whereas it partially formed on the surface of AG-CU. This difference in apatite formation ability between these samples is consistent with the intensity of the TF-XRD peak of apatite at the 2*θ* angle of around 32° being much smaller for AG-CU than for AG. The relationship between apatite formation ability in an SBF and the surface structure of TiO_2_ formed on Ti [13,31,38,39,40,41] or TiO_2_ gels [42] is not fully understood; nevertheless, in this study, the higher formation of anatase compared to that of rutile in AG (Figure 1b) may be responsible for the better apatite formation ability of AG.

**Figure 4:**
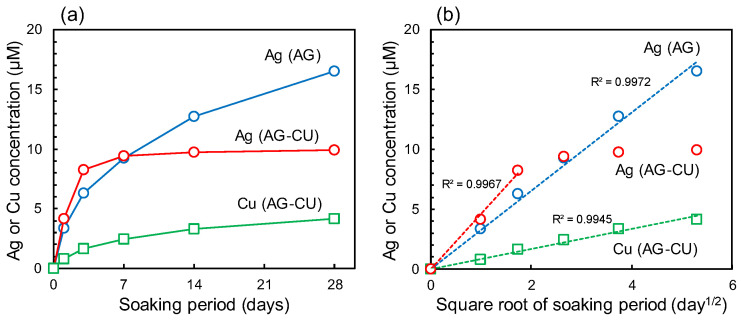
Ag and Cu ion release behavior from samples in PBS. (**a**) Accumulated-released amounts of Ag or Cu vs. soaking period and (**b**) accumulated-released amounts of Ag or Cu vs. square root of soaking period.

A slightly higher amount of Ag was released from AG-CU than from AG. The Ag concentration in PBS reached around 8 µM for 3 days (Figure 4a). However, the Ag concentration was saturated at around 7 days, which indicates that the release of Ag from AG-CU almost stopped at around 7 days. In contrast, AG released Ag gradually and continuously for 28 days. AG-CU slowly released Cu; the Cu concentration reached around 3 µM by day 28. These results indicate that AG-CU preferentially releases Ag over Cu, but the release of Ag is almost stopped at around 7 days even though AG continuously releases Ag for 28 days. Figure 4b was obtained by plotting the Ag and Cu concentrations against the square root of the soaking period. The concentration of Ag released from AG-CU within 3 days and that released from AG within 28 days are proportional to the square root of the soaking period. This result suggests that Ag was released from both samples via ion exchange [30,43], although the rate and duration of Ag release were different between the samples. The concentration of Cu released from AG-CU within 28 days is also proportional to the square root of the soaking period, which suggests that Cu was released from AG-CU via ion exchange [44]. However, the mechanism of Ag and Cu release from samples should be further investigated because Ag and Cu were mainly present as metallic silver with an oxidized surface and Cu_2_O, respectively (Figure 1 and Figure 2, and Table 2), and they are not likely to be released via ion exchange. The slightly more rapid release of Ag from AG-CU than from AG and the continuous release of Cu from AG-CU may lead to antibacterial activity that is somewhat strong at the initial stage of implantation and continues for a long period.

**Figure 5:**
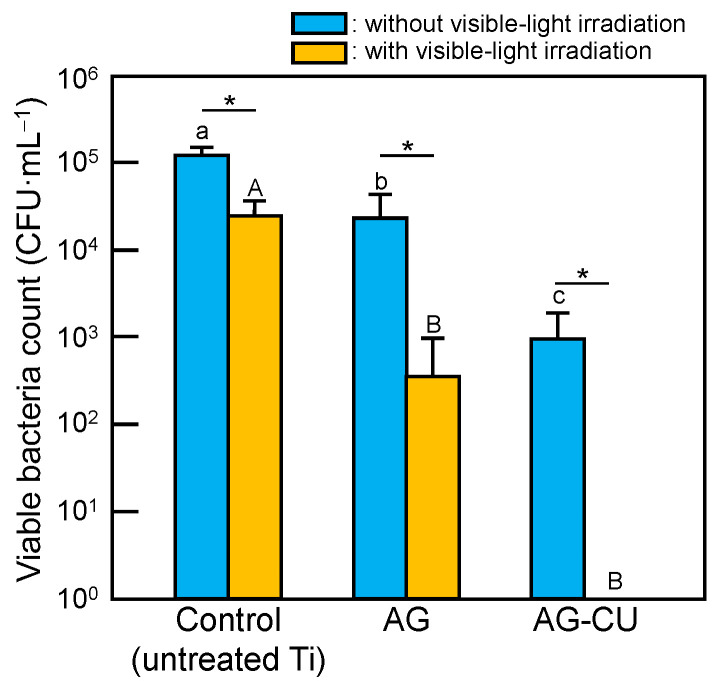
Number of viable bacteria for samples under conditions with and without visible-light irradiation. Bars with different letters (lowercase a–c for no visible-light irradiation group and uppercase A and B for visible-light irradiation group) are significantly different (*p* < 0.01). Asterisk (*) represents significant differences (*p* < 0.01) between no visible-light irradiation and visible-light irradiation.

Without visible-light irradiation, the number of viable bacteria was significantly smaller for AG and AG-CU than for untreated Ti, and slightly smaller for AG-CU than for AG (Figure 5). The rapid release of Ag and sustained release of Cu from AG-CU (Figure 4) might be responsible for the higher antibacterial activity of AG-CU compared to that of AG. The number of viable bacteria was significantly decreased by visible-light irradiation for AG-CU and AG compared to untreated Ti, and AG-CU showed extremely strong antibacterial activity under visible-light irradiation. The number of viable bacteria on untreated Ti (control) decreased under visible-light irradiation. Although an LED generates much less heat than a conventional incandescent bulb, the decrease in the number of viable bacteria on untreated Ti under visible-light irradiation may be attributed to the heat generated by the LED light, which was placed only 10 cm from the sample and had a high intensity of 250 W·m^−2^. Here, we briefly discuss the changes in the oxidation state of copper after antibacterial activity testing. Although XPS spectra of AG-CU after antibacterial testing should be measured to clarify the change in oxidation state of copper in the future, it is possible that Cu^2+^ is formed from Cu_2_O on the surface of AG-CU after antibacterial testing because the proportion of Cu^2+^ on the surface of copper metal increases after soaking in a bacteria-containing solution, and Cu^2+^ is the most stable chemical state against corrosion and bacteria [37].

Next, the antibacterial activity of AG-CU under visible-light irradiation is discussed in terms of the generation of ROS. The concentration of the hydroxyl radical (•OH) was measured via ESR using DMPO as the spin-trapping agent. Peaks of DMPO-OH were observed for AG-CU and AG. The intensity of the peaks was larger for AG-CU than for AG (Figure S1). Table 3 shows the concentrations of H_2_O_2_ and •OH for the samples. The H_2_O_2_ concentrations for all samples were less than 0.1 µM, much lower than the H_2_O_2_ concentrations (>1.25 µM) that can effectively kill *E. coli* [45,46,47]. The •OH concentration was higher for AG-CU than for AG and the control. Therefore, •OH radicals are likely to be generated by a reaction between hydroxide ions (OH^−^) and holes (h^+^), OH^−^ + h^+^ → •OH, namely a direct photocatalytic effect, on the surface of AG-CU. The generated •OH may contribute to the antibacterial activity of AG-CU under visible-light irradiation. In summary, it is thought that the excellent antibacterial activity of AG-CU under visible-light irradiation (Figure 5) can be attributed to the synergistic effect of the release of Ag and Cu (Figure 4) and the generation of •OH from the sample (Table 3). The details of the synergistic effect are still unclear, but it is possible that bacteria damaged by released Ag and Cu are more likely to be killed by •OH, or vice versa.

## 3. Materials and Methods

### 3.1. Sample Preparation

A commercially pure Ti chip with dimensions of 10 mm × 10 mm × 1 mm (purity: 99.9%, TIE04CB, Kojundo Chemical Lab. Co., Ltd., Saitama, Japan) was used as the original substrate and polished using a diamond pad (no. 400, Maruto Instrument Co., Ltd., Tokyo, Japan). The polished Ti chip was ultrasonically washed once with acetone (99%, Nacalai Tesque, Inc., Kyoto, Japan) and twice with ultrapure water for 10 min. The washed chip was dried at room temperature and atmospheric pressure. Subsequently, an aqueous NaOH solution was prepared by dissolving 1.031 g of NaOH (FUJIFILM Wako Pure Chemical Corp., Osaka, Japan) in 5 mL of ultrapure water. The washed chip was immersed in the NaOH aqueous solution in a round-bottomed polytetrafluoroethylene (PTFE) test tube with a cap (code 04936, SANPLATEC Corp., Osaka, Japan). The test tube was shaken at 120 strokes·min^−1^ for 24 h at 60 °C using a shaking bath. After the completion of the NaOH treatment, the Ti chip was removed from the test tube and washed with ultrapure water to obtain the NaOH-treated Ti chip. Subsequently, 0.085 g of silver nitrate (AgNO_3_, FUJIFILM Wako Pure Chemical Corp.) was dissolved in 5 mL of ultrapure water. The AgNO_3_ solution was diluted 100-fold to obtain approximately 1 mol·m^−3^ of AgNO_3_ solution. In addition, 0.121 g of Cu(NO_3_)_2_∙3H_2_O (FUJIFILM Wako Pure Chemical Corp.) was dissolved in 5 mL of ultrapure water. The Cu(NO_3_)_2_ solution was diluted 100-fold to obtain approximately 1 mol·m^−3^ of Cu(NO_3_)_2_ solution. A total of 3 mL of the diluted AgNO_3_ solution was mixed with 3 mL of the diluted Cu(NO_3_)_2_ solution and transferred to a round-bottomed PTFE test tube with a cap. The NaOH-treated Ti chip was then immersed in this mixture and shaken at 120 strokes·min^−1^ for 48 h at 80 °C. After the treatment, the chip was removed and washed with ultrapure water. The Ti chip treated with the AgNO_3_-Cu(NO_3_)_2_ mixed solution was heat-treated at 600 °C for 1 h using a muffle furnace (MSFS-1218, Yamada Denki Co., Ltd., Tokyo, Japan). The samples thus obtained are denoted as AG-CU. As a reference, the NaOH-treated Ti chips were immersed in 6 mL of the diluted AgNO_3_ solution in a round-bottomed PTFE test tube with a cap, and then heat-treated at 600 °C for 1 h. The samples thus obtained are denoted as AG.

### 3.2. Surface Structure Analysis

The surface morphology of the samples was observed using scanning electron microscopy (SEM; VE8800, Keyence Corp., Osaka, Japan). The crystalline phase of the surface layer formed by the solution and heat treatments was characterized using thin-film X-ray diffraction (TF-XRD; RINT2200VL, Rigaku Corporation, Tokyo, Japan) with Cu Kα radiation. The composition of the surface layer was evaluated using X-ray photoelectron spectroscopy (XPS; JPS-9010MC, JEOL, Tokyo, Japan). The X-ray source was monochromatic Mg Kα radiation (1253.6 eV) at 10 kV and 10 mA. The binding energy was calibrated using the C 1s photoelectron peak at 285.0 eV as a reference. XPS peak analysis was performed using CasaXPS (version 2.3.24, Casa Software Ltd., Devon, UK). The Shirley background was subtracted from all spectra prior to fitting. The surface composition was calculated from the XPS spectra using relative sensitivity factors obtained from the CasaXPS software library (C 1s, 1.0, O 1s, 2.93; Ti 2p_3/2_, 5.22; Ag 3d_5/2_ 10.68, Cu 2p_3/2_ 16.73). In addition, the modified Auger parameters (α’) of Ag and Cu were calculated from the Ag 3d_5/2_ and Ag M_4_VV peaks and from the Cu 2p_3/2_ and Cu L_3_VV peaks, respectively.

### 3.3. Evaluation of Apatite Formation Ability

The apatite formation ability of samples was evaluated using an SBF [48] that contained ions at concentrations (Na^+^: 142.0 mM; K^+^: 5.0 mM; Ca^2+^: 2.5 mM; Mg^2+^: 1.5 mM; Cl^−^: 147.8 mM; HCO_3_^−^: 4.2 mM; HPO_4_^2−^: 1.0 mM; SO_4_^2−^: 0.5 mM) nearly identical to those found in human blood plasma. The SBF was prepared according to the ISO 23317:2014 protocol. All chemicals used in the preparation of the SBF were purchased from Nacalai Tesque, Inc., Kyoto, Japan. An amount of 30 mL of the prepared SBF was poured into a centrifuge tube (ECK-50ML-R, AS-ONE Corp., Osaka, Japan). The samples were immersed in the SBF and kept at 36.5 °C. After 7 days, the samples were removed from the SBF, gently washed with ultrapure water, and dried at approximately 25 °C and atmospheric pressure. The lower surface of each sample was subjected to surface analysis using SEM and TF-XRD.

### 3.4. Ag and Cu Ion Release Behavior

To investigate the Ag and Cu ion release behavior of each sample, 10 mL of phosphate-buffered saline (PBS, 166-23555, FUJIFILM Wako Pure Chemical Corp.) was placed in a centrifuge tube (ECK-50ML-R, AS-ONE Corp.). The sample (*n* = 3) was immersed in PBS at 36.5 °C. The PBS was refreshed at appropriate periods. The accumulated and released amounts of Ag and Cu ions from the samples at 1, 3, 7, 14, and 28 days were calculated based on the Ag and Cu concentrations in the PBS, respectively, which were measured using inductively coupled plasma atomic emission spectroscopy (ICP-AES, iCAP600, Thermo Fisher Scientific Co., Ltd., Kanagawa, Japan).

### 3.5. Evaluation of Antimicrobial Activity

A nutrient agar was used in petri dishes (Falcon^®^ plastic dish for general bacteria, Corning Inc., New York, NY, USA) in 15 mL aliquots. Physiological saline was prepared by dissolving 8.5 g of sodium chloride (NaCl, Nacalai Tesque, Inc.) into 1 L of ultrapure water, which was used after sterilization at 121 °C for 20 min using a high-pressure steam sterilizer. *Escherichia coli* (*E. coli*, JCM5491) was used as the test bacterial strain. It was used after being cultured on the nutrient agar medium at 37 °C for 24 h. The bacterial mass of the cultured *E*. *coli* was taken with a platinum loop and dispersed in physiological saline to prepare a stock bacterial suspension (≅10^8^ CFU·mL^−1^). This stock suspension was diluted with a nutrient liquid medium to obtain a test bacterial suspension (≅10^7^ CFU·mL^−1^). The bacterial test was carried out for each sample (*n* = 4). A cell strainer (Corning Inc.) attached to a 6-well plate was used for setting the sample. The sample was placed on the cell strainer with the sample surface facing upward and 10 µL of the test bacterial suspension was dropped onto the sample. Subsequently, the sample surface was covered with a plastic film (9 mm × 9 mm × 0.06 mm) to achieve close contact. To reduce the effects of increasing temperature and drying during visible-light irradiation on the bacteria, a cooler was placed behind the 6-well plate and 1.5 mL of pure water was added to the wells to prevent the sample from drying. LED light (460 nm; SPA-10SW, Hayashi Clock Industry Co., Ltd., Tokyo, Japan) was used as the light source. The distance from the lower part of the lens to the sample surface was 10 cm, the irradiance was 250 W·m^−2^, and the irradiation period was 30 min. This irradiation period was set under the assumption of visible-light irradiation to the abutment of dental implants during dental treatments. As a control experiment, an antibacterial test without visible-light irradiation was also conducted. A schematic illustration of the antimicrobial activity evaluation system is shown in our previous paper [30]. After either irradiation with visible light for 30 min or no irradiation for 30 min, the sample was collected together with the film, soaked in 2 mL of soybean-casein digest broth with lecithin and polysorbate 80 (SCDLP, Nihon Pharmaceutical Co. Ltd., Osaka, Japan) medium, and thoroughly stirred to wash out the bacteria. The washed-out medium was diluted 10- and 100-fold with the SCDLP medium, and 100 µL of each was seeded onto the nutrient agar medium. These media were cultured at 37 °C for 48 h. Then, the number of colonies was counted and the viable cell count was calculated. The viable bacteria count for the AG-CU and AG groups was compared by performing a one-way analysis of variance and conducting a multiple-hypothesis test (Holm’s method).

### 3.6. Identification of Reactive Oxygen Species Induced by Visible-Light Irradiation

It is difficult to directly measure highly reactive oxygen species (ROS) and free radicals at around 25 °C. Therefore, we measured these chemical species via electron spin resonance (ESR; JES-FA-100, JEOL Ltd., Tokyo, Japan) using a spin-trapping method. 5,5-Dimethyl-1-pyrroline-N-oxide (DMPO, Labotech Co., Tokyo, Japan) was used as the spin-trapping agent. The measurement conditions were as follows: microwave power of 4.0 mW; microwave frequency of 9428.954 MHz; magnetic width of 0.1 mT; field sweep width of ±5 mT; field modulation frequency of 100 kHz; modulation width of 0.1 mT; time constant of 0.03 s; and sweep time of 0.1 min. The samples were placed in a 24-well plate and 500 µL of DMPO solution (300 mM) was added. The samples immersed in the DMPO solution were irradiated with visible light for 30 min under the same conditions as those in the antibacterial property test using LED light. Subsequently, 200 µL of the DMPO solution, in which a sample was immersed, was removed and the ROS were measured using an ESR spectrometer. 4-Hydroxy-2,2,6,6-tetramethylpiperidine-1-oxyl (TEMPOL, Sigma Aldrich, St. Louis, MO, USA) was used to quantify the hydroxyl radicals. A control ESR spectrum was obtained from a solution without sample immersion and visible-light irradiation. The amount of hydrogen peroxide (H_2_O_2_), which is an ROS, was measured using H_2_O_2_ colorimetry. Two types of solution were used for this purpose. Solution 1 was prepared by mixing 6 mL of 100 mM sulfuric acid and dissolving 11.8 mg of ammonium iron (II) sulfate hexahydrate into 30 mL of pure water. Solution 2 was prepared by dissolving 9.1 mg of xylenol orange tetrasodium salt and 2.186 g of sorbitol into 30 mL of pure water. A calibration curve was prepared using solutions 1 and 2, and 8.821 M H_2_O_2_ solution. A sample was placed in a 24-well plate and immersed in 500 µL of pure water. After irradiation with visible light for 30 min under the same conditions as those in the antibacterial property test using LED light, 400 µL of the pure water, in which the sample was immersed, was removed and poured into a glass tube. Subsequently, 200 µL of solution 1 and 200 µL of solution 2 were added into the glass tube and mixed well. The glass tube was then maintained at approximately 25 °C for 45 min. The absorbance of the mixture solution at a wavelength of 560 nm was then measured using ultraviolet-visible spectrophotometry (GeneQuant 1300, Biochrom, Ltd., Cambridge, UK).

## 4. Conclusions

TiO_2_ co-doped with Cu and Ag was formed on the surface of Ti via NaOH-(Cu(NO_3_)_2_ and AgNO_3_) and heat treatments. The TiO_2_ co-doped with Cu and Ag formed apatite on its surface in an SBF and showed higher antibacterial activity than that of TiO_2_ doped with only Ag, especially under visible-light irradiation. The excellent antibacterial activity of TiO_2_ co-doped with Cu and Ag under visible-light irradiation might be caused by the synergistic effect of the release of Ag and Cu and the generation of •OH from the sample. The toxicity of the sample needs to be evaluated in future studies, but dental implants with such a TiO_2_ surface layer co-doped with Cu and Ag may reduce the risk of peri-implantitis.

## Figures and Tables

**Table 1:** Surface composition of samples (mean ± SD).

Sample	Composition (at.%)				
	O	Ti	Ag	Cu	C
AG-CU	59.2 ± 0.6	31.0 ± 0.8	2.5 ± 0.2	4.9 ± 0.6	2.3 ± 0.2
AG	59.0 ± 1.2	31.7 ± 0.7	6.3 ± 1.8	−	3.1 ± 0.3

−: not measured.

**Table 2:** Summary of binding energy (*E_B_*) and modified Auger parameter (α′) values of samples (mean ± SD).

Sample	Element	*E_B_* (eV)	α′ (eV)
AG-CU	Ag	368.4 ± 0.2	723.5 ± 0.4
	Cu	933.0 ± 0.2	1850.4 ± 0.2
AG	Ag	368.6 ± 0.3	723.3 ± 0.1

**Table 3:** Concentrations of hydrogen peroxide (H_2_O_2_) and hydroxyl radical (•OH) for samples.

Sample	Concentration (µM)	
	H_2_O_2_	•OH
AG-CU	8.0 × 10^−2^	2.5
AG	8.9 × 10^−2^	1.3
Control (untreated Ti)	8.9 × 10^−2^	1.4

## Data Availability

The data will be available on request.

## Supplementary Materials

The following supporting information can be downloaded at: https://www.mdpi.com/article/10.3390/molecules28020650/s1, Figure S1: Electron spin resonance (ESR) spectra of control, AG, and AG-CU samples.

## References

[1] Atieh M.A., Alsabeeha N.H.M., Faggion C.M., Duncan W.J. (2013). The frequency of peri-implant diseases: A systematic review and meta-analysis. J. Periodontol..

[2] Lee C.T., Huang Y.W., Zhu L., Weltman R. (2017). Prevalences of peri-implantitis and peri-implant mucositis: Systematic review and meta-analysis. J. Dent..

[3] Dreyer H., Grischke J., Tiede C., Eberhard J., Schweitzer A., Toikkanen S.E., Glockner S., Krause G., Stiesch M. (2018). Epidemiology and risk factors of peri-implantitis: A systematic review. J. Periodont. Res..

[4] Kotsakis G.A., Olmedo D.G. (2021). Peri-implantitis is not periodontitis: Scientific discoveries shed light on microbiome-biomaterial interactions that may determine disease phenotype. Periodontol. 2000.

[5] Salvi G.E., Cosgarea R., Sculean A. (2017). Prevalence and mechanisms of peri-implant diseases. J. Dent. Res..

[6] Asensio G., Vizquez-Lasa B., Rojo L. (2019). Achievements in the topographic design of commercial titanium dental implants: Towards anti-peri-implantitis surfaces. J. Clin. Med..

[7] Shimabukuro M. (2020). Antibacterial property and biocompatibility of silver, copper, and zinc in titanium dioxide layers incorporated by one-step micro-arc oxidation: A review. Antibiotics.

[8] Dong H., Liu H., Zhou N., Li Q., Yang G.W., Chen L., Mou Y.B. (2020). Surface modified techniques and emerging functional coating of dental implants. Coatings.

[9] Foster H.A., Ditta I.B., Varghese S., Steele A. (2011). Photocatalytic disinfection using titanium dioxide: Spectrum and mechanism of antimicrobial activity. Microbiol. Biotechnol..

[10] Banerjee S., Dionysiou D.D., Pillai S.C. (2011). Self-cleaning applications of TiO_2_ by photo-induced hydrophilicity and photocatalysis. Appl. Catal. B Environ..

[11] Etacheri V., Di Valentin C., Schneider J., Bahnemann D., Pillai S.C. (2015). Visible-light activation of TiO_2_ photocatalysts: Advances in theory and experiments. J. Photochem. Photobiol. C Photochem. Rev..

[12] Suketa N., Sawase T., Kitaura H., Naito M., Baba K., Nakayama K., Wennerberg A., Atsuta M. (2005). An antibacterial surface on dental implants, based on the photocatalytic bactericidal effect. Clin. Implant Dent. Relat. Res..

[13] Uchida M., Kim H.-M., Kokubo T., Fujibayashi S., Nakamura T. (2002). Effect of water treatment on the apatite-forming ability of NaOH-treated titanium metal. J. Biomed. Mater. Res..

[14] Fujibayashi S., Nakamura T., Nishiguchi S., Tamura L., Uchida M., Kim H.-M., Kokubo T. (2001). Bioactive titanium: Effect of sodium removal on the bone-bonding ability of bioactive titanium prepared by alkali and heat treatment. J. Biomed. Mater. Res..

[15] Sato S. (1986). Photocatalytic activity of NO_x_-doped TiO_2_ in the visible light region. Chem. Phys. Lett..

[16] Sato S., Nakamura R., Abe S. (2005). Visible-light sensitization of TiO_2_ photocatalysts by wet-method N doping. Appl. Catal. A-Gen..

[17] Mathew S., Ganguly P., Rhatigan S., Kumaravel V., Byrne C., Hinder S.J., Bartlett J., Nolan M., Pillai S.C. (2018). Cu-doped TiO_2_: Visible light assisted photocatalytic antimicrobial activity. Appl. Sci..

[18] Chen M., Wang H., Chen X., Wang F., Qin X., Zhang C., He H. (2020). High-performance of Cu-TiO_2_ for photocatalytic oxidation of formaldehyde under visible light and the mechanism study. Chem. Eng. J..

[19] Janczarek M., Kowalska E. (2017). On the origin of enhanced photocatalytic activity of copper-modified titania in the oxidative reaction systems. Catalysts.

[20] Kaur R., Pal B. (2015). Plasmonic coinage metal-TiO_2_ hybrid nanocatalysts for highly efficient photocatalytic oxidation under sunlight irradiation. New J. Chem..

[21] Liu M., Sunada K., Hashimoto K., Miyauchi M. (2015). Visible-light sensitive Cu(II)-TiO_2_ with sustained anti-viral activity for efficient indoor environmental remediation. J. Mater. Chem. A.

[22] Moniz S.J.A., Tang J. (2015). Charge transfer and photocatalytic activity in CuO/TiO_2_ nanoparticle heterojunctions synthesised through a rapid, one-pot, microwave solvothermal route. Chem. Cat. Chem..

[23] Huang L., Peng F., Wang H., Yu H., Li Z. (2009). Preparation and characterization of Cu_2_O/TiO_2_ nano–nano heterostructure photocatalysts. Catal. Commun..

[24] Liu L.M., Yang W.Y., Li Q., Gao S.A., Shang J.K. (2014). Synthesis of Cu_2_O nanospheres decorated with TiO_2_ nanoislands, their enhanced photoactivity and stability under visible light illumination, and their post-illumination catalytic memory. ACS Appl. Mater. Inter..

[25] Park D., Choi E.J., Weon K.-Y., Lee W., Lee S.H., Choi J.-S., Park G.H., Lee B., Byun M.R., Baek K. (2019). Non-invasive photodynamic therapy against-periodontitis-causing bacteria. Sci. Rep..

[26] Kawashita M., Matsui N., Miyazaki T., Kanetaka H. (2013). Effect of ammonia or nitric acid treatment on surface structure, in vitro apatite formation, and visible-light photocatalytic activity of bioactive titanium metal. Colloid Surf. B Biointerfaces.

[27] Kawashita M., Yokohama Y., Cui X.Y., Miyazaki T., Kanetaka H. (2014). In vitro apatite formation and visible-light photocatalytic activity of Ti metal subjected to chemical and thermal treatments. Ceram. Int..

[28] Kawashita M., Endo E., Watanabe T., Miyazaki T., Furuya M., Yokota K., Abiko Y., Kanetaka H., Takahashi N. (2016). Formation of bioactive N-doped TiO_2_ on Ti with visible light-induced antibacterial activity using NaOH, hot water, and subsequent ammonia atmospheric heat treatment. Colloid Surf. B Biointerfaces.

[29] Iwatsu M., Kanetaka H., Mokudai T., Ogawa T., Kawashita M., Sasaki K. (2020). Visible light-induced photocatalytic and antibacterial activity of N-doped TiO_2_. J. Biomed. Mater. Res. Part B.

[30] Suzuki K., Yokoi T., Iwatsu M., Mokudai T., Kanetaka H., Kawashita M. (2021). Antibacterial properties of Cu-doped TiO_2_ prepared by chemical and heat treatment of Ti metal. J. Asian Ceram. Soc..

[31] Kawashita M., Iwabuchi Y., Suzuki K., Furuya M., Yokota K., Kanetaka H. (2018). Surface structure and in vitro apatite-forming ability of titanium doped with various metals. Colloid Surf. A Physicochem. Eng. Asp..

[32] Kizuki T., Matsushita T., Kokubo T. (2014). Antibacterial and bioactive calcium titanate layers formed on Ti metal and its alloys. J. Mater. Sci. Mater. Med..

[33] Rajendran A., Pattanayak D.K. (2014). Silver incorporated antibacterial, cell compatible and bioactive titania layer on Ti metal for biomedical applications. RSC Adv..

[34] Rajendran A., Pattanayak D.K. (2020). Mechanistic studies of biomineralisation on silver incorporated anatase TiO_2_. Mater. Sci. Eng. C Mater. Biol. Appl..

[35] Hanaor D.A.H., Sorrell C.C. (2011). Review of the anatase to rutile phase transformation. J. Mater. Sci..

[36] Ferraria A.M., Carapeto A.P., do Rego A.M.B. (2012). X-ray photoelectron spectroscopy: Silver salts revisited. Vacuum.

[37] Shimabukuro M., Manaka T., Tsutsumi Y., Nozaki K., Chen P., Ashida M., Nagai A., Hanawa T. (2020). Corrosion behavior and bacterial viability on different surface states of copper. Mater. Trans..

[38] Takadama H., Kim H.-M., Kokubo T., Nakamura T. (2001). TEM-EDX study of mechanism of bonelike apatite formation on bioactive titanium metal in simulated body fluid. J. Biomed. Mater. Res..

[39] Wang X.-X., Hayakawa S., Tsuru K., Osaka A. (2002). Bioactive titania gel layers formed by chemical treatment of Ti substrate with a H_2_O_2_/HCl solution. Biomaterials.

[40] Yang B., Uchida M., Kim H.-M., Zhang X., Kokubo T. (2004). Preparation of bioactive titanium metal via anodic oxidation treatment. Biomaterials.

[41] Wei D., Zhou Y., Jia D., Wang Y. (2007). Characteristic and in vitro bioactivity of a microarc-oxidized TiO_2_-based coating after chemical treatment. Acta Biomater..

[42] Uchida M., Kim H.-M., Kokubo T., Fujibayashi S., Nakamura T. (2003). Structural dependence of apatite formation on titania gels in a simulated body fluid. J. Biomed. Mater. Res. Part A.

[43] Kawashita M., Toda S., Kim H.-M., Kokubo T., Masuda M. (2003). Preparation of antibacterial silver-doped glass microspheres. J. Biomed. Mater. Res. Part A.

[44] Bajpai S.K., Bajpai M., Sharma L. (2012). Copper nanoparticles loaded alginate-impregnated cotton fabric with antibacterial properties. J. Appl. Polym. Sci..

[45] Imlay J.A., Linn S. (1986). Bimodal pattern of killing of DNA-repair-defective or anoxically grown *Escherichia coli* by hydrogen peroxide. J. Bacteriol..

[46] Imlay J.A., Chin S.M., Linn S. (1988). Toxic DNA damage by hydrogen peroxide through the Fenton reaction in vivo and in vitro. Science.

[47] Linley E., Denyer S.P., McDonnell G., Simon C., Maillard J.-Y. (2012). Use of hydrogen peroxide as a biocide: New consideration of its mechanisms of biocidal action. J. Antimicrob. Chemother..

[48] Takadama H., Kokubo T. (2006). How useful is SBF in predicting in vivo bone bioactivity?. Biomaterials.

