# Supplementary figures and images for: Visible-Light-Enhanced Antibacterial Activity of Silver and Copper Co-Doped Titania Formed on Titanium via Chemical and Thermal Treatments
Source: Molecules. 2023 Jan 9;28(2):650. doi: 10.3390/molecules28020650 (PMC9866272; doi:10.3390/molecules28020650)

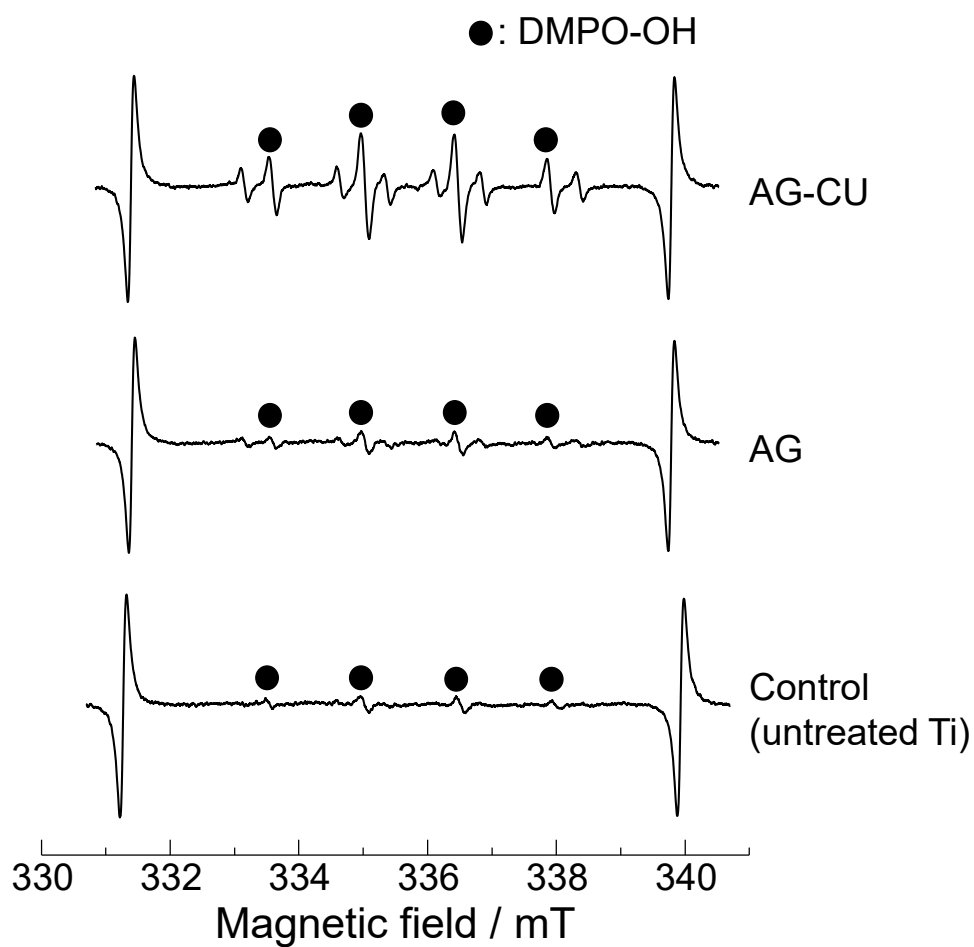

**Figure S1.** Electron spin resonance (ESR) spectra of control, AG, and AG-CU samples.

Supplement: Supplementary file 1 [file molecules-28-00650-s001.zip › molecules-2064142-supplementary.pdf]
